# SHROOM2 inhibits tumor metastasis through RhoA–ROCK pathway-dependent and -independent mechanisms in nasopharyngeal carcinoma

**DOI:** 10.1038/s41419-019-1325-7

**Published:** 2019-01-25

**Authors:** Jing Yuan, Lin Chen, Jingshu Xiao, Xue-Kang Qi, Ji Zhang, Xu Li, Zifeng Wang, Yi-Fan Lian, Tong Xiang, Yuchen Zhang, Ming-Yuan Chen, Jin-Xin Bei, Yi-Xin Zeng, Lin Feng

**Affiliations:** 1 Department of Experimental Research, Sun Yat-sen University Cancer Center, State Key Laboratory of Oncology in South China, Collaborative Innovation Center for Cancer Medicine, Guangdong Key Laboratory of Nasopharyngeal Carcinoma Diagnosis and Therapy, Guangzhou, 510060 China; 2 Department of Oncology, The First Affiliated Hospital of Zhengzhou University, Zhengzhou, 450052 China; 3 Department of Neurosurgery, Sun Yat-sen University Cancer Center, Guangzhou, 510060 China; 4 School of Life Sciences, Westlake University, Hangzhou, 310024 China; 5 Guangdong Province Key Laboratory of Liver Disease Research, The Third Affiliated Hospital of Sun Yat-sen University, Guangzhou, China; 6 Department of Nasopharyngeal Carcinoma, Sun Yat-sen University Cancer Center, Guangzhou, 510060 China

## Abstract

SHROOM2 is a key mediator of RhoA–ROCK pathway that regulates cell motility and actin cytoskeleton organization. However, the functions of SHROOM2 beyond RhoA/ROCK signaling remain poorly understood. Here, we report that SHROOM2 not only participates in RhoA–ROCK-induced stress fiber formation and focal adhesion, but also had an unanticipated role in suppressing epithelial-to-mesenchymal transition (EMT) and tumor metastasis. Depletion of SHROOM2 in nasopharyngeal carcinoma (NPC) cells enhances mesenchymal characteristics and reduces epithelial markers, concomitant with increased motility, enabling the development of invasion and tumor metastasis, which are largely ROCK-independent, as ROCK inhibitor Y-27632 did not cause EMT phenotype; furthermore, combination of ROCK inhibition and SHROOM2 depletion resulted in the most robust increases in cell migration and invasion, indicating that SHROOM2 and ROCK work synergistically rather than epistatic. Analysis of clinical samples suggested that SHROOM2 is downregulated in NPC and the expression of SHROOM2 in metastatic NPC was even lower than in the primary tumors. Our findings uncover a non-canonical role of SHROOM2 as a potent antagonist for EMT and NPC metastasis.

## Introduction

Metastasis is the leading cause of death in patients with solid tumors^1^, such is true in nasopharyngeal carcinoma (NPC), a common type of cancer in Southern China and Southeast Asia but is rare worldwide^2^. Although NPC tends to be more sensitive to radiotherapy than other head and neck cancers, around 10% of cases present distant metastasis after radiotherapy with or without concurrent chemotherapy, which accounts for the majority of deaths from NPC^3^.

A great effort has been devoted towards finding novel therapeutic approaches that would prevent tumor invasion and metastasis, or biomarkers which could predict the metastasis risk of cancer patients, however, limited success has been achieved, largely due to the complexity of metastasis process, which is sequentially executed by local invasion, intravasation, dissemination, extravasation and colonization, and the cancer cells must opt to different characteristics during the entire metastasis process^4^. In the past two decades, a process named epithelial-to-mesenchymal transition (EMT) has been proven as a key driver for cancer metastasis. EMT program converts epithelial cells into a more mesenchymal state, enabling epithelial cells to lose their cell-to-cell junctions and the apical-basal polarity while acquiring migratory and invasive capacities, facilitating escape from the primary tumor^5–7^. The essentiality of EMT for metastasis has been demonstrated in NPC^8^. However, other players in NPC metastasis other than the known EMT transcription factors have not been not fully characterized.

Rho GTPases is one of the most important families of proteins regulating the cell migration, which play crucial roles in the regulation of cell morphology, motility, cell–cell and cell–matrix adhesion. There are more than 20 members in this family, represented by RhoA, RAC1, and CDC42^9,10^. Rho Kinase (ROCK) is a key serine/threonine kinase downstream of Rho which mediating the formation of RhoA-induced stress fibers and focal adhesions through phosphorylation of downstream targets, like LIM kinase, myosin light chain (MLC) and Myosin Phosphatase-Targeting Subunit 1 (MYPT1)^11–13^. ROCK has been considered as an anti-cancer target because of its role in promoting invasion and migration of various types of cancer^14–16^. The mammalian ROCK family is composed of ROCK1 and ROCK2, and the two isoforms play similar roles in vivo^17^. Y-27632, a selective small inhibitor of both ROCK1 and ROCK2, has been shown to suppress the invasiveness and metastasis of rat and human hepatoma cells^18,19^, bladder cancer cells^20^, colorectal cancer cells^21^, lung cancer cells^22^, and others. However, ROCK inhibition by Y-27632 resulted in a dramatic stimulation in the invasion of colon cancer cell line on 3D culture system^23^, and a recent study showed that RhoA inactivation or Y-27632 treatment promotes migration and metastasis of triple-negative breast cancer^24^. Moreover, RhoA inactivation by expressing a dominant negative RhoA (T19N) increases tumor growth and metastasis in colorectal and liver cancers^25,26^. The contradictory effects of Rho–ROCK pathway inhibition restrict the application of Y-27632 to anti-cancer therapy and more studies are needed to fully address the regulation of Rho–ROCK signaling pathway in tumor metastasis.

SHROOM2 belongs to SHROOM family which consists of four different yet closely related proteins, which share a PDZ domain and two conserved SD (SHROOM domain) domains, SD1 and SD2. The SD2 domain binds to ROCK directly and mediates its activation^27–29^. *SHROOM2* has been reported to be associated with the susceptibility or carcinogenesis of esophageal squamous carcinoma and colorectal cancer^30,31^, however, the exact role of SHROOM2 in the development of cancer and whether it works solely downstream of ROCK have not been fully elucidated. To answer this question, we investigated the role of SHROOM2 in NPC. Our results suggest that SHROOM2 is downregulated in NPC and is implicated in the suppression of cancer cell invasion and metastasis by preventing EMT, which is largely independent of Rho–ROCK signaling. These results provide novel insights into the mechanism by which SHROOM2 participates in tumor suppression.

## Results

### Downregulation of SHROOM2 in NPC

**Fig. 1:**
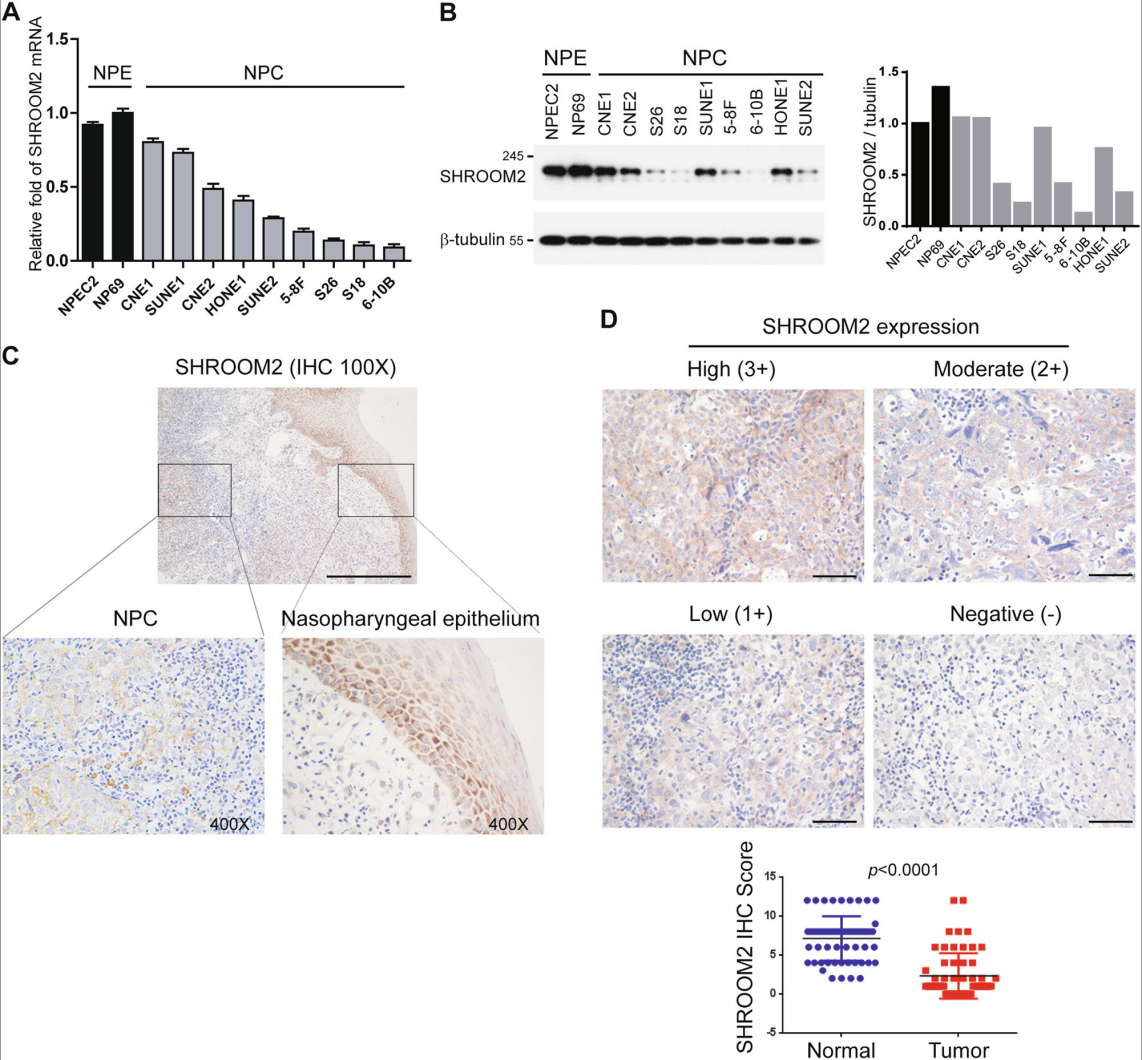
SHROOM2 is downregulated in NPC cell lines and tumor tissues. **a** Quantitative PCR analysis of SHROOM2 expression in NPE and NPC cell lines. **b** (left) Western blot analysis of SHROOM2 expression in cell lines as in **a** with β-tubulin as the loading control. (right) Quantification of western blot with ImageJ. **c** Immunohistochemical (IHC) image of NPC sample containing both normal adjacent epithelial and tumor tissues stained with an anti-SHROOM2 antibody, together with an enlarged view of each in the corresponding inset. Scale bar: 100 μm. **d** (top) Immunohistochemistry staining of SHROOM2 in NPC biopsies. Scale bar: 50 μm. (bottom) Scatterplots representing the IHC scores of SHROOM2 in 60 NPC tissues and paired adjacent normal nasopharynx tissues (two-tailed unpaired *t*-test)

We first evaluated SHROOM2 expression in normal nasopharyngeal epithelial (NPE) cell lines and nasopharyngeal carcinoma (NPC) cell lines. Quantitative PCR and Western blot analyses suggested that both mRNA and protein levels of SHROOM2 were lower in NPC cell lines than in NPE cell lines NPEC2 and NP69 (Fig. 1a, b). To elucidate the status of SHROOM2 in NPC clinical samples, in situ immunohistochemistry (IHC) in 60 NPC and matched normal nasopharynx tissues were performed. In normal nasopharyngeal epithelium adjacent to the tumor, SHROOM2 staining was homogenous in all cells of the basal layer and suprabasal layers and appeared to be less intense in the more differentiated layers (Fig. 1c, right panel). In contrast, SHROOM2 expression was low in NPC tissues (Fig. 1c, left panel). The intensity of SHROOM2 staining in NPC was scored on a gray scale as negative (−), low (1+), moderate (2+), and high (3+) (Fig. 1d, top panel). Scoring of SHROOM2 IHC based on staining strength and the percent of positive cells revealed that SHROOM2 expression was significantly downregulated in human NPC compared to non-cancerous nasopharyngeal tissues (Fig. 1d, bottom panel). These results indicated that SHROOM2 might act as a suppressor in NPC development.

### SHROOM2 suppresses migration and invasion of NPC cells

**Fig. 2:**
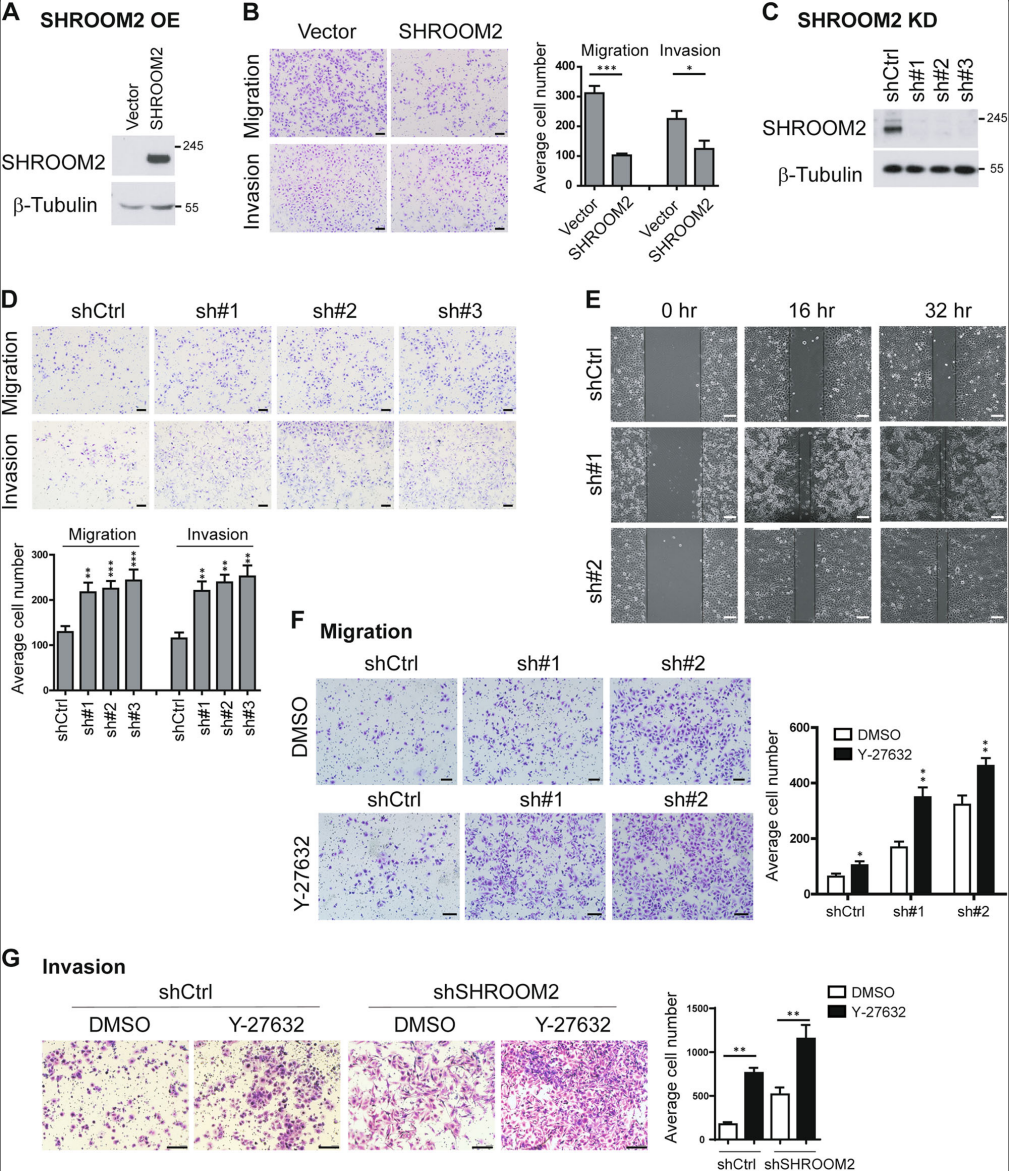
SHROOM2 suppresses cell migration and invasion. **a** Immunobloting of SHROOM2 in HONE1 cells that stably expressing pBABE-SHROOM2. **b** Representative images (left) and quantification (right) of Transwell cell migration and invasion assays for HONE1 cells that stably expressing ectopic SHROOM2 or empty vector. Scale bar: 100 μm. Mean ± SD, *n* = 3. **c** Western blot analysis of HONE1 cells infected with control lentivirus or lentivirus carrying SHROOM2-specific shRNAs. **d** Representative images (top) and quantification (bottom) of Transwell cell migration and invasion assays for control HONE1 cells or HONE1 cells with SHROOM2 knockdown by three independent shRNAs. Scale bar: 100 μm. Mean ± SD, *n* = 3. **e** Wound healing assays for control and SHROOM2 knockdown HONE1 cells. Scale bar: 100 μm. **f** Representative images (left) and quantification (right) of Transwell cell migration assays for control and SHROOM2 knockdown HONE1 cells treated with 20 μM Y-27632 or DMSO for 24 h. Scale bar: 50 μm. Mean ± SD, *n* = 3. **g** Transwell invasion assays as in **f** except the upper chamber was coated with Matrigel. Scale bar: 50 μm. *p < 0.05, **p < 0.01, ***p < 0.001, two-tailed unpaired *t*-test

To investigate the role of SHROOM2 in cancer development, we overexpressed SHROOM2 in HONE1, a typical NPC cell line with characteristic of poorly differentiated squamous cell carcinomas^32^ (Fig. 2a). Cell migration and invasion were determined using non-matrigel-coated or matrigel-coated transwell cell culture chambers, respectively and the results suggested SHROOM2 overexpression significantly inhibited cell migration and invasion (Fig. 2b). Conversely, silencing of SHROOM2 by three SHROOM2-specific shRNAs in HONE1 cells all led to evident increases in migration and invasion (Fig. 2c, d). In addition, scratch wound healing assay also supported a role of SHROOM2 in suppressing cell migration as quicker closure of the scratched “wound” were observed in cells lacking SHROOM2 (Fig. 2e).

SHROOM2 has been characterized as a mediator of RhoA–ROCK pathway. To investigate if SHROOM2 inhibits cell migration through ROCK signaling, HONE1 cells with or without SHROOM2 depletion were treated with DMSO or ROCK inhibitor Y-27632. Surprisingly, compare with the strong induction of migration by SHROOM2 knockdown, only a slight increase in the proportion of migrated cells was observed by Y-27632 treatment. Furthermore, migration of SHROOM2-depleted cells was further promoted by adding ROCK inhibitor Y-27632 (Fig. 2f). Consistently, invasion assay also obtained similar results (Fig. 2g). These data indicate that SHROOM2 restricts cell migration and invasion through additional mechanism other than ROCK signaling.

### SHROOM2 suppresses metastasis of NPC

**Fig. 3:**
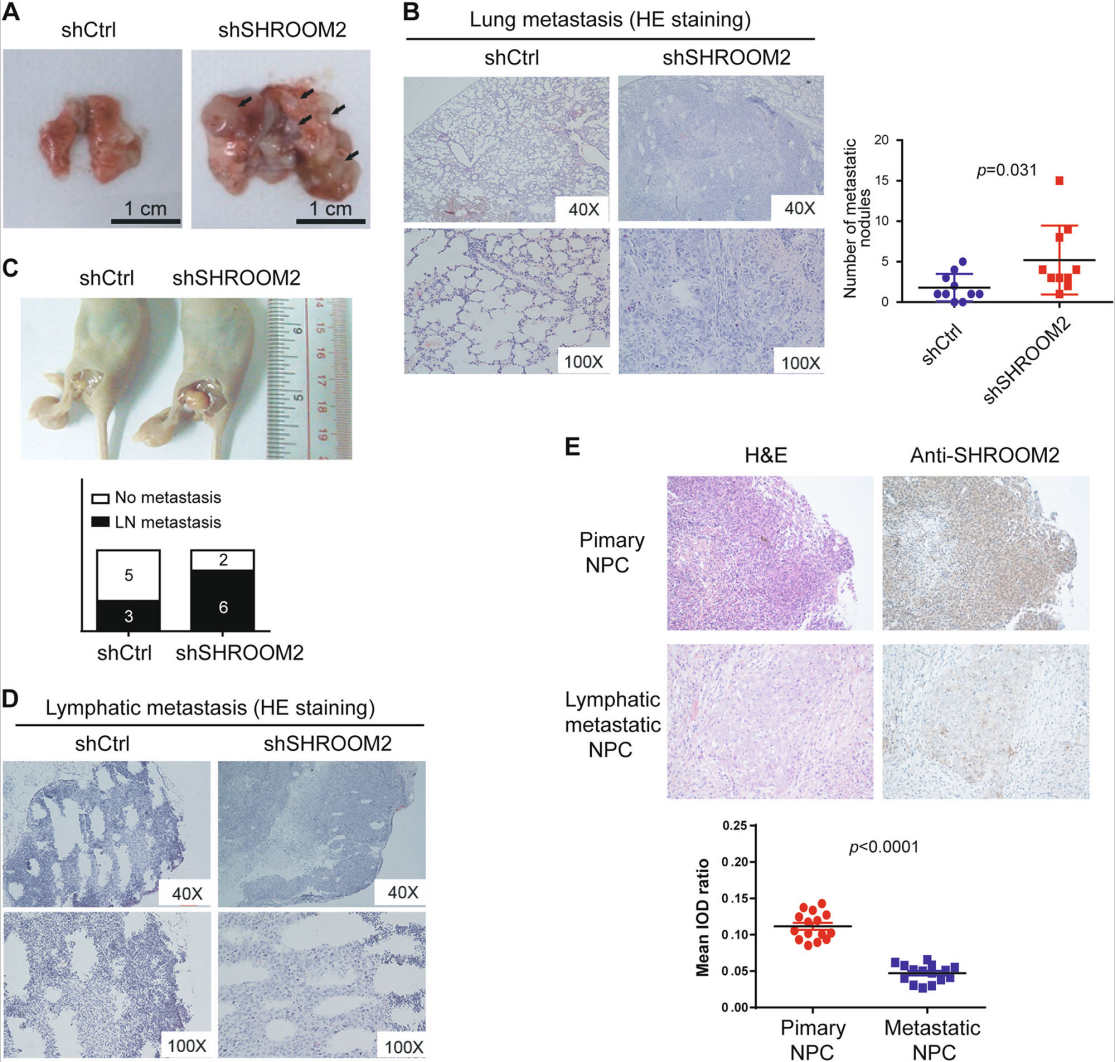
SHROOM2 inhibits NPC metastasis. **a** Metastatic nodule on the lung surface driven by tail vein injection of control and SHROOM2 knockdown HONE1 cells. **b** (left) Lung sections stained by hematoxylin and eosin (H&E) showed more metastatic nodules in SHROOM2 knockdown cells than in control cells. (right) Number of metastatic nodules formed in lung of mice injected with control and SHROOM2 knockdown cells, *n* = 10, two-tailed unpaired *t*-test. **c** Representative images (top) and quantification (bottom) of mice with lymph node (LN) metastasis. Control or SHROOM2 knockdown HONE1 cells were injected into the left hind foodpat of athymic mice, 7 weeks later the LN metastasis were determined by naked eye and H&E staining (**d**), *n* = 8. **e** (top) Representative images of H&E and IHC staining for SHROOM2 in 15 paired primary and metastatic NPC tumor samples. (bottom) Tissue image analysis with Image-Pro Plus 6.0 according to the average integrated optical density (IOD) per stained area (μm^2^) (IOD/area). Five independent images taken from each group were analyzed, and the results are shown as the means ± SD (two-tailed unpaired *t*-test)

Having established the suppressive role of SHROOM2 in cell migration and invasion, we then examined the role of SHROOM2 in metastasis by tail vein injection of cancer cells, which primarily form metastatic nodules in the lung. At 7 weeks after injection, mice were sacrificed, and the lungs were harvested. The number of metastatic nodules was significantly higher in mice injected with SHROOM2 knockdown cells than in the mice injected with control NPC cells (Fig. 3a, b; *p* = 0.031).

To further elucidate the anti-migratory and anti-invasive functions of SHROOM2, a spontaneous lymph node metastasis mouse model was established by injecting SHROOM2 knockdown NPC cells and control cells into the left hind footpad of immune-deficient nude mice, which resulting in metastasis to the popliteal lymph node. Consistent with the results from tail vein injection, cells with SHROOM2 depletions had a significantly greater potential of lymphatic metastasis (Fig. 3c, d).

Next, we evaluated SHROOM2 expression in 15 paired primary/metastatic NPC samples. IHC staining revealed that the metastatic NPC tumors had lower levels of SHROOM2 compared to the primary tumors (Fig. 3e), further supporting a suppressive role of SHROOM2 in NPC metastasis in vivo. On the other side, we also determined the proliferation rate of control and SHROOM2 knockdown cells, but no significant difference was observed (Supplementary Fig. 1a). Furthermore, SHROOM2-depleted NPC cells exhibited a similar rate of tumor growth compared with control cells in xenograft assay (Supplementary Fig. 1b**;** *p*>0.05). Taken together, SHROOM2 mainly regulates metastatic capacity but not tumorigenicity of NPC.

### SHROOM2 maintains epithelial morphology

**Fig. 4:**
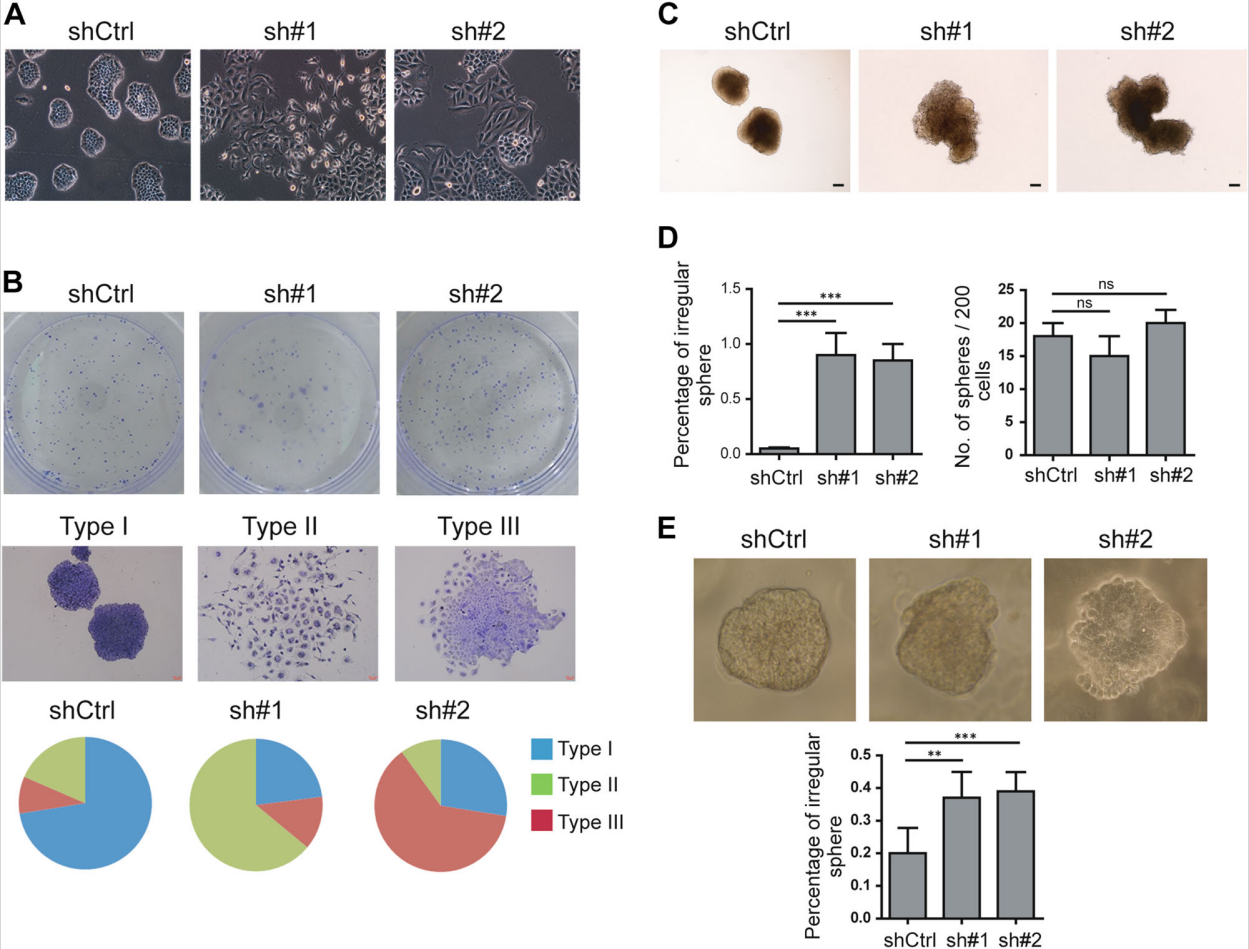
SHROOM2 is required for epithelial morphology maintenance. **a** Morphology of control and SHROOM2 knockdown HONE1 cells observed by bright field microscopy. **b** (top) Colonies formed by control and SHROOM2 knockdown HONE1 cells on conventional tissue culture plates. (middle) Representative colony images as classified with Type I, II and III. (bottom) Quantification of different types of colonies formed by control and SHROOM2 knockdown cells. **c** Morphological features of control and SHROOM2-depleted HONE1 cells in soft agar culture. Scale bar: 50 μm. **d** Quantification of spheres formed in **c** with irregular shapes (left) and total number of the spheres (right). **e** (top) Morphology and (bottom) quantification of spheres with irregular shape of control and SHROOM2-depleted HONE1 cells grown in Matrigel. ns not significant, *p < 0.05, **p < 0.01, ***p < 0.001, two-tailed unpaired *t*-test

SHROOM family is implicated in morphogenesis of native epithelial cells during development^33^, we then examined the effect of SHROOM2 depletion on NPC cell morphology. HONE1 cells maintain highly organized cell–cell adhesion and cell polarity, while knockdown of SHROOM2 led to loss of cell–cell contacts and cell scattering: a cobble-stone-like morphology was replaced with a spindle-like, fibroblastic appearance upon SHROOM2 depletion (Fig. 4a). In the colony formation assay, the majority of control HONE1 cells formed tight and regular colonies as classified as type I, with less colonies that exhibited appearance with loosely packed cells (type II) or a mosaic appearance (type III); in contrast, SHROOM2-depleted group produced more type II and type III colonies compared with the control group (Fig. 4b).

In line with the morphology in 2D culture condition, HONE1 cells formed tightly packed spheres in soft agar, while spheres formed by SHROOM2-depleted cells were looser and more irregular (Fig. 4c, d, left). There were no significant differences in the number of spheres formed in control group and SHROOM2-silencing groups (Fig. 4d, right). When cultured in Matrigel, HONE1 control cells formed well-polarized spheres, while SHROOM2-depleted cells lost the polarity and became invasive in the matrix (Fig. 4e).

### Loss of SHROOM2 induces EMT

**Fig. 5:**
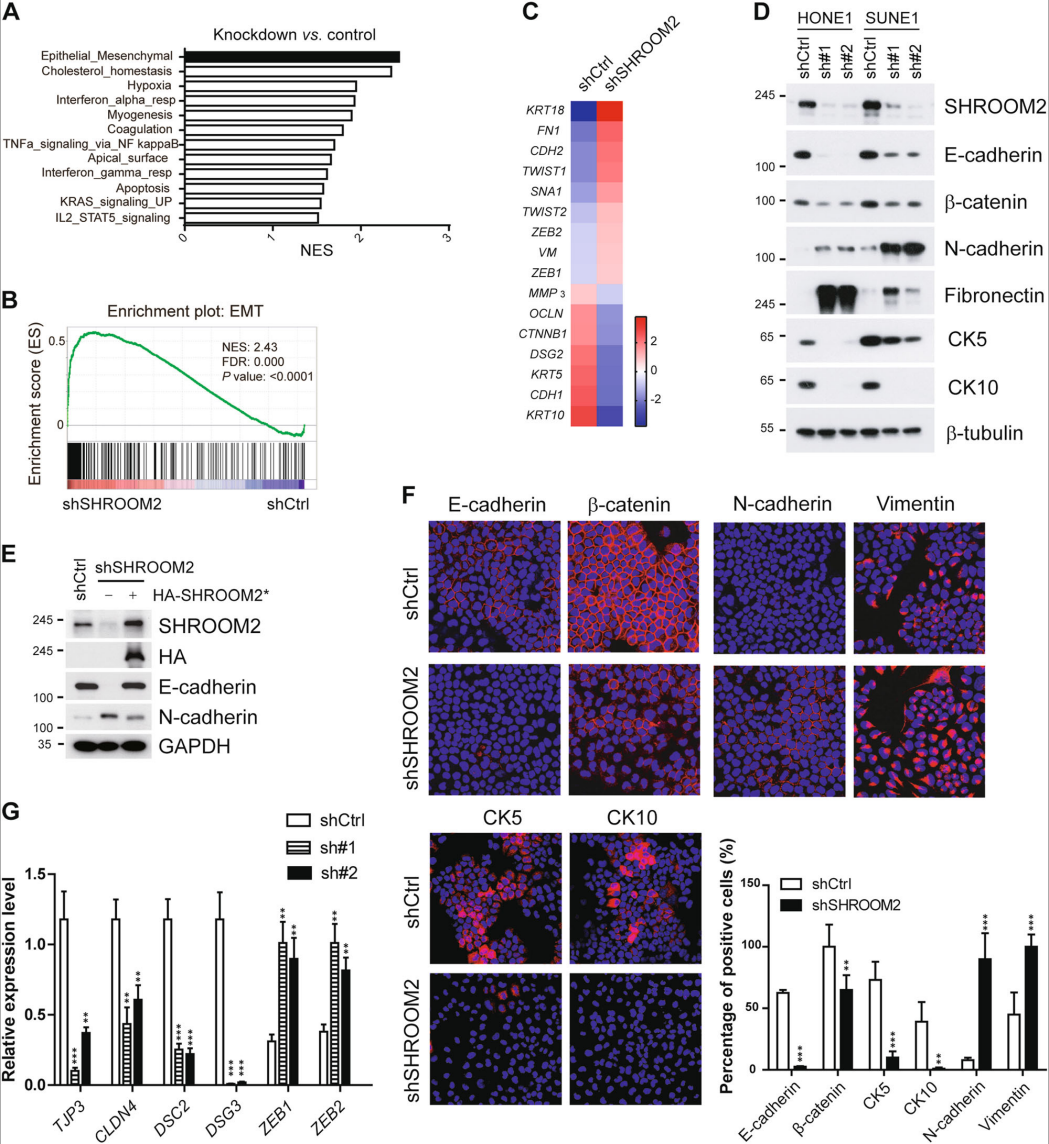
SHROOM2 inhibits EMT. **a** Gene set enrichment analysis (GSEA) of microarray data of control and SHROOM2 knockdown HONE1 cells. NES normalized enrichment score. **b** GSEA plot showing an enrichment of gene signatures associated with EMT between control and SHROOM2 knockdown HONE1 cells; FDR: false-discovery rate. **c** Heat map of the EMT-related genes up- and downregulated by SHROOM2 depletion from the microarray data. The scale is −2 to +2 in log 10. **d** Western blot analysis of EMT markers in HONE1 and SUNE1 cells infected with lentivirus carrying control shRNA or shRNAs targeting SHROOM2. **e** Reconstitution of SHROOM2 into SHROOM2-depleted HONE1 cells rescued E- and N-cadherin expression. Asterisk denotes shRNA-resistant SHROOM2. **f** Immunofluorescence staining of EMT markers (top) and keratins (bottom, left) in control and SHROOM2-depleted HONE1 cells. Quantification of the percentage of the positive staining was shown (bottom, right). Experiments were performed in triplicate and at least 100 cells were counted in each experiment. **g** qPCR analysis of cell–cell junction molecules and EMT markers in control and SHROOM2-depleted cells. Mean ± SD, *n* = 3. *p < 0.05, **p < 0.01, ***p < 0.001, two-tailed unpaired *t*-test

To uncover the mechanisms underlying the inhibition of SHROOM2 on NPC cell migration and metastasis, an analysis of the whole transcriptome by microarray was conducted in HONE1 cells with or without SHROOM2 depletion. Functional profiling of the differentially expressed genes suggested that the greatest proportion was enriched in EMT (Fig. 5a), a process where epithelial cells take on characteristics of mesenchymal cells and play key roles in tumor metastasis and development. Gene set enrichment analysis (GSEA) suggested that SHROOM2 knockdown cells displayed a more mesenchymal trait than the control cells (Fig. 5b). We profiled changes in EMT-related gene expression upon SHROOM2 depletion and the heat map showed an upregulation of mesenchymal markers and downregulation of epithelial markers (Fig. 5c). Indeed, depletion of SHROOM2 by two shRNAs in two NPC cell lines, HONE1 and SUNE1 led to EMT as evidenced by downregulation of epithelial markers E-cadherin and β-catenin and upregulation of mesenchymal markers N-cadherin and fibronectin (Fig. 5d). Additionally, we performed a rescue experiment by reconstituting SHROOM2-depleted cells with an shRNA-resistant SHROOM2. The downregulation of E-cadherin and upregulation of N-cadherin in the depleted cells were reversed by the reconstituted SHROOM2 (Fig. 5e), indicating that SHROOM2 exerts a specific effect on EMT. Besides, the microarray data revealed several adhesion molecules were significantly downregulated by loss of SHROOM2, such as tight junction proteins TJP3 (Tight Junction Protein 3) and CLDN4 (Claudin 4), desmosome proteins DSC2 (Desmocollin 2) and DSG3 (Desmoglein 3), and intermediate filament proteins CK5 (Keratin 5) and CK10 (Keratin 10), which were verified by immunofluorescence assay (Fig. 5f) and quantitative PCR (Fig. 5g). These data indicate that SHROOM2 is involved in the suppression of EMT.

### SRHOOM2 regulates stress fibers and focal adhesions in a ROCK-dependent manner, but inhibits EMT independently of ROCK pathway

**Fig. 6:**
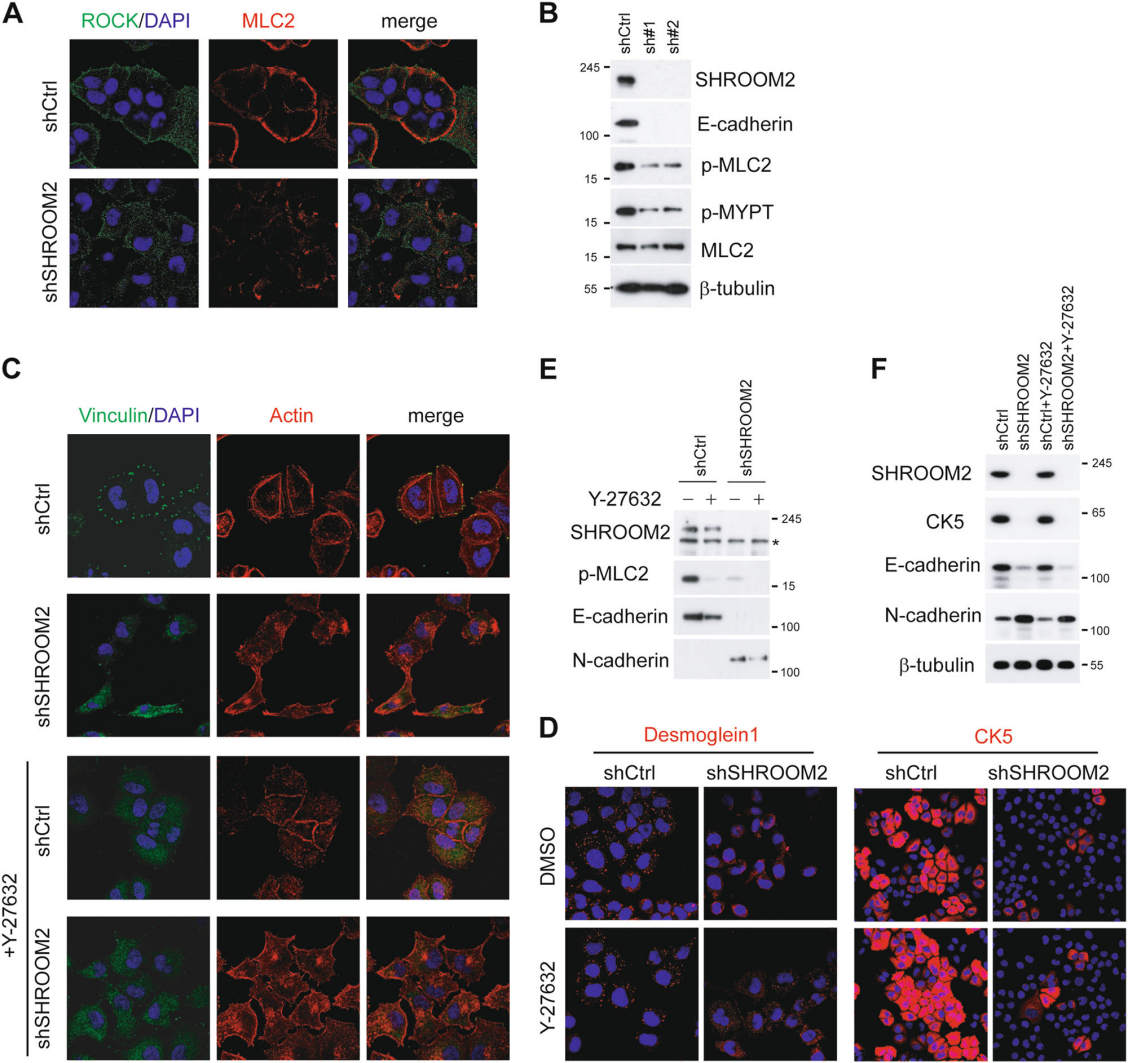
SHROOM2 regulates stress fibers and focal adhesions via ROCK pathway but inhibits EMT independently of ROCK. **a** Immunofluorescence staining of ROCK and MLC2 in control and SHROOM2-depleted HONE1 cells. **b** Immunoblotting of phosphorylated MLC2 and MYPT in control and SHROOM2-depleted HONE1 cells. **c** Subcellular localization of Vinculin and Actin in control and SHROOM2-depleted cells treated with 20 μM Y-27632 or DMSO for 24 h. **d** Immunostaining of Desmoglein1 and CK5 in control and SHROOM2 knockdown HONE1 cells with or without Y-27632 treatment. **e** WB analysis of phospho-MLC2 and EMT markers in control and SHROOM2-depleted HONE1 cells with or without Y-27632 treatment as in **c**. **f** WB analysis of CK5 expression as in **e**

SHROOM2 has been characterized as a ROCK binding protein that mediating ROCK membrane recruitment and activation^27,28,34^, we next tried to elucidate whether the anti-EMT function of SHROOM2 is ROCK pathway-dependent. First, we examined the subcellular distribution of ROCK and myosin regulatory light chain (MLC2), a substrate of ROCK in control and SHROOM2-depleted cells. Consistent with previous reports, loss of SHROOM2 greatly impaired membrane recruitment of ROCK1 and MLC2 (Fig. 6a). Correspondingly, phosphorylation of ROCK substrates, MLC2 and MYPT were significantly decreased upon SHROOM2 depletion (Fig. 6b). Next, we determined the importance of SHROOM2 in RhoA–ROCK-mediated stress fibers and focal adhesions (FAs) formation. HONE1 cells displayed long organized stress fibers as visualized by phalloidin-fluorescence and formed discernible focal adhesions (FAs) as labeled by anti-Vinculin antibody, Vinculin is an adaptor protein linking actin stress fibers to integrin at the plasma membrane. When the cells were treated with ROCK inhibitor Y-27632, destruction of stress fibers and FAs were observed. On the other hand, depletion of SHROOM2 reduced actin polymerization and FAs formation to a similar extent as that seen for control cells treated with Y-27632, and addition of Y-27632 to SHROOM2 knockdown cells did not lead to appreciable further reduction on stress fibers and FAs formation (Fig. 6c). Besides, ROCK inhibition did not further reduce desmosome formation as demonstrated by Desmoglein1 (DSG1) and CK5 immunostaining (Fig. 6d). On the other side, Y-27632 treatment had no effects on E- and N-cadherin (Fig. 6e) and CK5 (Fig. 6f) expression, indicating that the anti-EMT function of SHROOM2 is independent of RhoA–ROCK pathway, while its role in stress fiber formation and focal adhesion is ROCK-dependent. Taken together, SHROOM2 suppresses migration and metastasis of NPC cells in both ROCK-dependent and independent manner, the latter is implicated in EMT regulation.

## Discussion

Cell migration is central to metastasis. Rho–ROCK pathway has been linked to tumor cell metastasis by regulating actin rearrangement and focal adhesions within cells. ROCK is the most well characterized downstream effector of Rho, which is activated through several mechanisms, including binding of GTP-bound form of Rho to the Rho-binding domain (RBD) of ROCK^35,36^, lipid binding to the PH domain^37^, and binding of SHROOM to the SHROOM-binding domain (SBD) of ROCK^28,29^. Activated ROCK interacts with the actin cytoskeleton to induce stress fiber formation and assembly of focal contacts by phosphorylatin myosin light chain^12,38^. Inhibition of ROCK pathway has been shown to suppress metastasis in multiple types of cancer^18–22^. However, contrasting reports have been published on the role of RhoA–ROCK pathway in tumor invasion and metastasis^23–26^. In this study, we found that Y-27632 treatment resulting in increased NPC cell migration and invasion (Fig. 2f and g), concomitant with the reduced MLC phosphorylation, stress fiber and focal adhesion formation (Fig. 6). The cell type and context-dependent effect of Y-27632 on cell migration might result from the complex outcome of ROCK inhibition. On one hand, disruption of F-actin bundles by ROCK inhibition impairs cell contractility, therefore Y-27632 often leads to decreased cell migration and invasion as observed in various cancer cells^18–22^; On the other hand, stress fibers are necessary for the maintenance of cell–cell and cell–ECM adhesion, and losing adhesion in fact facilitates the early step in metastatic dissemination, which may account for the increased migration of cancer cells as observed in this study and other reports^23–26^. It is conceivable that the balance between the changes in contractility and adhesion dictates the net effect of Y-27632. The notion has been further supported by the data showing that loss of SHROOM2, which resulted in a reduction in the levels of adhesion proteins like E-cadherin, tight junction proteins and desmosome proteins (Fig. 5), further accelerates cell migration and invasion than single ROCK inhibitor treatment (Fig. 2f, g). Thus, our data established a new paradigm for ROCK inhibition in metastasis prevention, indicating the cellular environment should be taken into consideration when use ROCK inhibitors as anti-tumor agents.

SHROOM family consists of four isoforms, SHROOM1-4. SHROOM family members have similar domain architecture, with SHROOM-domain 1 (SD1) and SHROOM-domain 2 (SD2) that bind F-actin and ROCK, respectively. SHROOM use the SD2 domain to recruit ROCK to the cytoskeletion which in turn phosphorylates non-muscle myosin II to remodel cellular morphology; meanwhile, SHROOM-binding directly activates ROCK kinase by relieving autoinhibitory conformation of ROCK^28,29^. To date, the well-known functions of SHROOM are all linked to ROCK pathway. In this study, we found that SHROOM2 works independently of ROCK in controlling EMT and migration. SHROOM2 depletion, but not ROCK inhibition was able to induce EMT and EMT-associated cell migration and invasion. Loss of SHROOM2 not only impedes ROCK activation, as characterized by the reduced stress fiber and FA assembly, but also shows clear phenomenon of EMT, such as a switch to mesenchymal-like morphology, downregulation of E-cadherin and upregulation of N-cadherin, all are hallmarks of EMT. As ROCK inhibitor Y-27632 does not affect the expression of these EMT markers, which is in line with a prior report^39^, and addition of Y-27632 further increases the motility of SHROOM2-depleted cells (Fig. 2f), indicating that SHROOM2 and ROCK work synergistically rather than epistatic. Whether the anti-EMT function is unique to SHROOM2 or also applicable to other SHROOM isoforms needs further investigation.

SHROOM2 loss may result in the activation of specific downstream signal transduction pathways that eventually leads to EMT, which confers traits on cancer cells facilitating completion metastasis process. EMT is driven by multiple EMT transcription factors that includes ZEB1/2, Twist, Slug and OVOL2^5,8^. In the microarray data, we found that ZEB1 and ZEB2 levels were markedly increased in SHROOM2-depleted cells (Fig. 5e). At present, we are unclear what downstream signal transduced by SHROOM2 that directly controlling EMT process. Recently, *SHROOM2* mutations have been found in patients with neural tube defect (NTD) in Chinese cohort. Interestingly, none of these point mutations cause appreciable reduction in the interaction with ROCK^40^. Neural tube closure is one of the best studied development example of EMT^41^, it is possible that these mutations abrogate the association of SHROOM2 with a unknown player in EMT program. However, purification of SHROOM2 in 293T cells did not capture any proteins that play definite role in EMT, although ROCK protein was obtained (Supplementary table 1). Future studies on SHROOM2-intereacting protein network by developing approaches suitable for capturing transient and week protein–protein interactions would be helpful for elucidating the mechanisms by which SHROOM2 antagonizes EMT.

In summary, our study uncovered an anti-metastatic role of SHROOM2 through inhibition of EMT. More complete understanding of how SHROOM and RhoA–ROCK pathway coordinately and specifically regulate cancer cell metastasis may throw light on the therapeutic approaches for advanced NPC.

## Materials and methods

### Cell culture

The NPC cell lines (CNE1, CNE2, S18, S26, SUNE1, 5–8 F, 6-10B, SUNE1 and HONE1) were cultured in Dulbecco’s modified Eagle medium (Gibco) with 10% FBS. The immortalized nasopharyngeal epithelium (NPE) cell lines NPEC2-Tert and NP69 were cultured in keratinocyteserum-free growth medium supplemented with 5 μg/L EGF and 50 mg/L Bovine Pituitary Extract (Gibco) as described previously^8,42^. SUNE1 and HONE1 have cell line authentication report using short tandem repeart (STR) profiling.

### shRNAs, stable knockdown, and reconstitution of shRNA-resistant SHROOM2

ShRNAs targeting SHROOM2 were cloned in pLKO.1-puro vector (Sigma). The shRNA sequences are as follows: control shRNA: 5′-TTCAATAAATTCTTGAGGTTT-3′; three SHROOM2 targeting shRNAs: 1# 5′-GATGAGATCGTCGGCATCAAT-3′, 2# 5′-GAGCGCATCGTCTTTGACATT-3′. 3# 5′-CCACCAATTCTACCTACTACA-3′. Stable knockdown cells were established by puromycin (2 μg/mL) selection as previously described^43^. SHROOM2 shRNA-resistant construct was generated by introducing silent mutations (5′-GAGCGCAT*T*GT*T*TT*C*GA*T*AT*C*-3′) in the shSHROOM2 #2 targeting sequence using PCR-mediated site-directed mutagenesis and then subcloned into the lentiviral vector pLVX-IRES-hygro. Rescue experiment was performed by transduction of shRNA-resistant pLVX-HA-SHROOM2 into SHROOM2-knockdown cells and selected with hygromycin (200 μg/mL).

### Establishment of stable cell lines

To generate SHROOM2 stable cell line, SHROOM2 cDNA was cloned into the pBABE-puro vector and packaged into lentivirus by co-transfecting of packaging plasmids pMD2G and pSPAX2 into HEK293T cells using polyethylenimine (PEI; 25 kDa) method. Forty-eight hours after transfection, the supernatant was collected and used for infection. Stable pools were selected with medium containing 2 μg/mL puromycin (Sigma). Empty pBABE vector was used as control.

### Microarray analysis

Total RNA from HONE1 control and SHROOM2 knockdown cells was isolated with TRIzol reagent (Invitrogen). Human Genome U133 Plus 2.0 Arrays (Affymetrix) were used for gene expression analysis by CapitalBio Corporation (Beijing, China).

### Quantitative real-time PCR

Total RNA was isolated with TRIzol reagent (Invitrogen) according to the manufacturer’s instructions. First-strand cDNA was synthesized using a PrimeScript RT Reagent Kit (TaKaRa). Quantitative real-time PCR was performed using Platinum SYBR Green qPCR SuperMix (Invitrogen) according to the manufacturer’s instructions. The PCR primers used in this study are as follows: SHROOM2-F: 5′-CCTGAAGCTGGTCGTCAAAAG-3′, SHROOM2-R: 5′- CGCTGTAGGTTCGTCTGCT-3′;TJP3-F:5′- GCTTTGGCATTGCGATCTCTG-3′, TJP3-R:5′- GATGTGGTCGCCTGTCTGTAG-3′;CLDN4-F:5′-TGGGGCTACAGGTAATGGG-3′, CLDN4-R:5′-GGTCTGCGAGGTGACAATGTT-3′;DSC2-F:5′-CCAATTCCTTGTTCGATGCTAGA-3′, DSC2-R:5′-GGCCGTGTCAGATTGAACC-3′:DSG3-F:5′-GCAAAAACGTGAATGGGTGAAA-3′, DSG3-R:5′-TCCAGAGATTCGGTAGGTGATT-3′;E-cadherin-F: 5′-TGCCCAGAAAATGAAAAAGG-3′, E-cadherin-R: 5′-GTGTATGTGGCAATGCGTTC-3′; N-cadherin-F: 5′’-AGCCAACCTTAACTGAGGAGT-3′, N-cadherin-R: 5′-GGCAAGTTGATTGGAGGGATG-3’; Vimentin-F: 5′-GAGAACTTTGCCGTTGAAGC-3′, Vimentin-R: 5′-GCTTCCTGTAGGTGGCAATC-3′; ZEB1-F: 5′-ATGCAGCTGACTGTGAAGGT-3′, ZEB1-R: 5′-GAAAATGCATCTGGTGTTCC-3′; ZEB2-F: 5′-GTCCATGCGAACTGCCATCTGATCCGCTCT-3′, ZEB2-R: 5′-GGCTTGCAGAATCTCGCCAC-3′; CK5-F: 5′-CAGAGCCACCTTCTGCGTCCTG-3′, CK5-R: 5′-GCTGAAGCTACGACTGCCCCC-3′; CK10-F: 5′-GGTGGGAGTTATGGAGGCAG-3′, CK10-R: 5′-CGAACTTTGTCCAAGTAGGAAGC-3′; β-catenin-F: 5′-GCTACTGTTGGATTGATTCGAAATC-3′, β-catenin-R: 5′-CCCTGCTCACGCAAAGGT-3′ and GAPDH-F: 5′-TTGCCATCAATGACCCCTTCA-3′, GAPDH-R: 5′-CGCCCCACTTGATTTTGGA-3′.

### Antibodies

The following antibodies were used in this study: SHROOM2 (SAB2700199), Vinculin (V9131) and Phalloidin–Tetramethylrhodamine B isothiocyanate (P1951) were from Sigma; E-Cadherin (24E10), N-Cadherin (13116), MLC2 (3672), p-MLC2 (T18/S19) (3674), HA (3724) were from Cell Signaling Technology; ROCK1 (ab58305), CK5 (ab52635), CK10 (ab76318), Desmoglein1 (ab12077) and RAC1 (ab155938) were from abcam; p-MYPT (ABS45) was from Millipore and Fibronectin (610077) was from BD biosciences. Horseradish peroxidase-conjugated goat anti-mouse/rabbit secondary antibodies were purchased from Promega.

Immunofluorescence microscopy, cell proliferation, colony formation, soft agar assays were performed as described previously^8,42^.

### Migration and invasion assays

Cell migration and invasion were analyzed using non-matrigel-coated transwell chambers (8 μm pore size polycarbonate filter) (FALCON) and matrigel-coated invasion chamber (Corning), respectively. Briefly, cells were resuspended in 200 μL DMEM medium (4 × 10^4^ for HONE1 cells and 8 × 10^4^ for SUNE1 cells) and plated in the upper insert of a 24-well chamber. Cells were incubated for 20 h at 37 °C and cells on the upper side of the filters were removed by scrubbing with a cotton swab. The cells on the bottom surface of the membrane were subsequently fixed with methanol for 10 min and stained with crystal violet dye for 30 min. Migrated and invasive cells were counted in five randomly chosen fields using an inverted microscope (OLYMPUS, IX71).

### Scratch wound assay

6 × 10^5^ SUNE1 cells and 7.5 × 10^5^ HONE1 cells were seeded in a six-well plate, respectively. After culturing for 24 h, cells were scratched with a 10 μL tip pipette to generate consistently sized wounds. Cells at the same wound site were photographed at the times indicated after scratching.

### 3D culture assay

Cells from each group were collected and resuspended in Matrigel at a concentration of 5 × 10^3^ per 50 μL, and plated into the center of each well in 24-well plates. Culture medium was composed of the basal medium-advanced DMEM/F12 and supplements including GlutaMAX, B27 serum-free supplement, N2 supplement (Life technologies), 10 mM HEPES and 1 mM *N*-Acetyl-l-cysteine (Sigma). Cells were then covered with culture medium and grown for 6–9 days, changing medium every 2 days.

### Tissue samples and immunohistochemistry

The human study was approved by Ethic Committees of Sun Yat-sen University Cancer Center (Protocol No. YB2013-04). This study was conducted under the provisions of the Declaration of Helsinki and informed written consents were obtained from all patients. A total of 60 specimens of NPC tissue with normal tissue adjacent to the tumors were obtained from Sun yat-sen university cancer center (SYSUCC, Guangzhou, China). Tissue samples that had been histologically and clinically confirmed were obtained from the archives of the Department of Sample Resources, Sun Yat-sen University Cancer Center. For metastasis study, an additional cohort of 15 patient samples with paired primary and metastatic tumors were collected. Among them, 12 patients with lymphatic metastasis and 3 with liver metastasis.

Immunohistochemistry of the paraffin-embedded sections was performed as previously described^42^. The sections were incubated overnight at 4 °C with rabbit anti-SHROOM2. The degree of immunostaining was evaluated and scored independently by two pathologists, and the staining intensity and proportion of positively stained tumor cells were used as evaluation criteria. The tumor cell proportion was scored as follows: 0 (no positive tumor cells), 1 (≤30% positive tumor cells), 2 (31–50% positive tumor cells), 3 (51–75% positive tumor cells), and 4 (≥76% positive tumor cells). The staining intensity was graded according to the following criteria: 0 (no staining), 1 (weak staining, light yellow), 2 (moderate staining, yellowish brown), and 3 (intense staining, brown). The staining index was calculated by multiplying the above two scores to yield a final score of 0, 1, 2, 3, 4, 6, 8, 9, or 12.

### Animal studies

All the animal experiments were performed with the approval of Institutional Animal Care and Use Committee of Sun Yat-sen University (reference no. GZR2016-105) and the animals were handled in accordance with institutional guidelines. Female BALB/c nude mice of 5-week-old were purchased from Shanghai Laboratory Animal Center were chosen randomly to perform in vivo assay. For tumorigenicity assay, 1 × 10^6^ SHROOM2 stable knockdown cells or control cells were injected subcutaneously into the right dorsal flank of each mouse, respectively. Tumor volume (V) was measured every two days and calculated with the formula *V* = (length × width^2^)/2.

For tail vein assay of metastasis, SHROOM2 knockdown cells or control cells (1 × 10^6^ cells) were injected intravenously though the tail vein into each nude mouse. All mice were sacrificed 8 weeks after inoculation. Lungs were formalin-fixed, paraffin-embedded, then sectioned 4 μm thick and stained with H&E.

Spontaneous lymph node metastasis assay was performed as described previously. Briefly, 2 × 10^5^ cancer cells were injected s.c. into the footpad of left hind limb of each nude mouse. After seven weeks observation, the mice were sacrificed and the popliteal lymph nodes from both hind limbs were isolated using a dissecting microscope.

### Affinity purification of SHROOM2

HEK293T cells stably expressing SFB (S-Flag-Streptavidin-binding peptide) -tagged SHROOM2 were amplified and subjected to TAP according to our previous methods^43,44^. Mass spectrometry (MS) were performed by Beijing Proteome Research Center. The MS list of SHROOM2-interacting proteins can be found in Supplementary Table 1.

### Statistical analysis

Data were analyzed with the GraphPad Prism 6 software. Two-tailed Student’s *t*-test was used to compare the statistical differences between groups. All *p* values <0.05 were considered statistically significant.

## Supplementary information

Supplementary Figure

Supplementary Table 1

## Data Availability

The transcriptome has been deposited in the NCBI’s Gene Expression Omnibus (GEO) (http://www.ncbi.nlm.nih.gov/geo) with the accession number GSE119020.

## References

[1] Friberg S, Nystrom A (2015). Cancer metastases: early dissemination and late recurrences. Cancer Growth Metastas-..

[2] Chua MLK, Wee JTS, Hui EP, Chan ATC (2016). Nasopharyngeal carcinoma. Lancet.

[3] Wei KR (2014). Nasopharyngeal carcinoma incidence and mortality in China in 2010. Chin. J. Cancer.

[4] Wells A, Grahovac J, Wheeler S, Ma B, Lauffenburger D (2013). Targeting tumor cell motility as a strategy against invasion and metastasis. Trends Pharmacol. Sci..

[5] Nieto MA, Huang RY, Jackson RA, Thiery JP (2016). EMT: 2016. Cell.

[6] Lo HC, Zhang XH (2018). EMT in metastasis: finding the right balance. Dev. Cell.

[7] Zhang, Y. & Weinberg, R. A. Epithelial-to-mesenchymal transition in cancer: complexity and opportunities. *Front. Med*. **12**, 361–373 (2018). 10.1007/s11684-018-0656-6 PMC6186394 30043221

[8] Qi XK (2018). OVOL2 links stemness and metastasis via fine-tuning epithelial-mesenchymal transition in nasopharyngeal carcinoma. Theranostics.

[9] Ellenbroek SI, Collard JG (2007). Rho GTPases: functions and association with cancer. Clin. Exp. Metastas-..

[10] Tang Y, Olufemi L, Wang MT, Nie D (2008). Role of Rho GTPases in breast cancer. Front. Biosci..

[11] Kimura K (1996). Regulation of myosin phosphatase by Rho and Rho-associated kinase (Rho-kinase). Science.

[12] Amano M (1997). Formation of actin stress fibers and focal adhesions enhanced by Rho-kinase. Science.

[13] Riento K, Ridley AJ (2003). Rocks: multifunctional kinases in cell behaviour. Nat. Rev. Mol. Cell Biol..

[14] Amano M, Nakayama M, Kaibuchi K (2010). Rho-kinase/ROCK: a key regulator of the cytoskeleton and cell polarity. Cytoskeleton.

[15] Campbell H (2018). 133p53 isoform promotes tumour invasion and metastasis via interleukin-6 activation of JAK-STAT and RhoA-ROCK signalling. Nat. Commun..

[16] Mu G (2018). Gastrin stimulates pancreatic cancer cell directional migration by activating the Galpha12/13-RhoA-ROCK signaling pathway. Exp. Mol. Med..

[17] Thumkeo D, Shimizu Y, Sakamoto S, Yamada S, Narumiya S (2005). ROCK-I and ROCK-II cooperatively regulate closure of eyelid and ventral body wall in mouse embryo. Genes Cells.

[18] Itoh K (1999). An essential part for Rho-associated kinase in the transcellular invasion of tumor cells. Nat. Med..

[19] Takamura M (2001). Inhibition of intrahepatic metastasis of human hepatocellular carcinoma by Rho-associated protein kinase inhibitor Y-27632. Hepatology.

[20] Jiang L, Wen J, Luo W (2015). Rhoassociated kinase inhibitor, Y27632, inhibits the invasion and proliferation of T24 and 5367 bladder cancer cells. Mol. Med. Rep..

[21] Voorneveld PW (2014). Loss of SMAD4 alters BMP signaling to promote colorectal cancer cell metastasis via activation of Rho and ROCK. Gastroenterology.

[22] Li B (2006). Involvement of Rho/ROCK signalling in small cell lung cancer migration through human brain microvascular endothelial cells. FEBS Lett..

[23] Vishnubhotla R, Bharadwaj S, Sun S, Metlushko V, Glover SC (2012). Treatment with Y-27632, a ROCK inhibitor, increases the proinvasive nature of SW620 cells on 3D collagen type 1 matrix. Int. J. Cell Biol..

[24] Humphries B (2017). ARHGAP18 downregulation by miR-200b suppresses metastasis of triple-negative breast cancer by enhancing activation of RhoA. Cancer Res..

[25] Rodrigues P (2014). RHOA inactivation enhances Wnt signalling and promotes colorectal cancer. Nat. Commun..

[26] Chew TW (2014). Crosstalk of Ras and Rho: activation of RhoA abates Kras-induced liver tumorigenesis in transgenic zebrafish models. Oncogene.

[27] Farber MJ, Rizaldy R, Hildebrand JD (2011). Shroom2 regulates contractility to control endothelial morphogenesis. Mol. Biol. Cell.

[28] Mohan S (2012). Structure of Shroom domain 2 reveals a three-segmented coiled-coil required for dimerization, Rock binding, and apical constriction. Mol. Biol. Cell..

[29] Zalewski JK (2016). Structure of the Shroom-Rho kinase complex reveals a binding interface with monomeric shroom that regulates cell morphology and stimulates kinase activity. J. Biol. Chem..

[30] Timme S (2014). STAT3 expression, activity and functional consequences of STAT3 inhibition in esophageal squamous cell carcinomas and Barrett’s adenocarcinomas. Oncogene.

[31] Dunlop MG (2012). Common variation near CDKN1A, POLD3 and SHROOM2 influences colorectal cancer risk. Nat. Genet..

[32] Glaser R (1989). Two epithelial tumor cell lines (HNE-1 and HONE-1) latently infected with Epstein-Barr virus that were derived from nasopharyngeal carcinomas. Proc. Natl Acad. Sci. USA.

[33] Lee C, Scherr HM, Wallingford JB (2007). Shroom family proteins regulate gamma-tubulin distribution and microtubule architecture during epithelial cell shape change. Development.

[34] Nishimura T, Takeichi M (2008). Shroom3-mediated recruitment of Rho kinases to the apical cell junctions regulates epithelial and neuroepithelial planar remodeling. Development.

[35] Ishizaki T (1996). The small GTP-binding protein Rho binds to and activates a 160 kDa Ser/Thr protein kinase homologous to myotonic dystrophy kinase. EMBO J..

[36] Matsui T (1996). Rho-associated kinase, a novel serine/threonine kinase, as a putative target for small GTP binding protein Rho. EMBO J..

[37] Araki S (2001). Arachidonic acid-induced Ca2+sensitization of smooth muscle contraction through activation of Rho-kinase. Pflug. Arch..

[38] Ishizaki T (1997). p160ROCK, a Rho-associated coiled-coil forming protein kinase, works downstream of Rho and induces focal adhesions. FEBS Lett..

[39] Sahai E, Marshall CJ (2002). ROCK and Dia have opposing effects on adherens junctions downstream of Rho. Nat. Cell Biol..

[40] Chen Z, Kuang L, Finnell RH, Wang H (2018). Genetic and functional analysis of SHROOM1-4 in a Chinese neural tube defect cohort. Hum. Genet..

[41] Thiery JP, Acloque H, Huang RY, Nieto MA (2009). Epithelial-mesenchymal transitions in development and disease. Cell.

[42] Li N (2018). BET bromodomain inhibitor JQ1 preferentially suppresses EBV-positive nasopharyngeal carcinoma cells partially through repressing c-Myc. Cell Death Dis..

[43] Zhang HJ (2018). Epstein-Barr virus activates F-box protein FBXO2 to limit viral infectivity by targeting glycoprotein B for degradation. PLoS Pathog..

[44] Deng C (2018). TNFRSF19 inhibits TGFbeta signaling through interaction with TGFbeta receptor type I to promote tumorigenesis. Cancer Res..

