# Supplementary material for: SHROOM2 inhibits tumor metastasis through RhoA–ROCK pathway-dependent and -independent mechanisms in nasopharyngeal carcinoma
Source: Cell Death Dis. 2019 Jan 25;10(2):58. doi: 10.1038/s41419-019-1325-7 (PMC6347642; doi:10.1038/s41419-019-1325-7)

## Supplementary Fig. 1 SHROOM2 does not regulate cell proliferation and tumorigenicity of NPC. a Cell proliferation of control and SHROOM2 knockdown HONE1 (left) and SUNE1 (right) cells by two shRNAs as determined by CCK-8 assay. b (left) Control and SHROOM2 knockdown HONE1 cells were subcutaneously injected into nude mice, and images were taken 15 days post-implantation. (right) Growth curves of xenograft tumors formed by control and SHROOM2 knockdown HONE1 cells (n = 9).


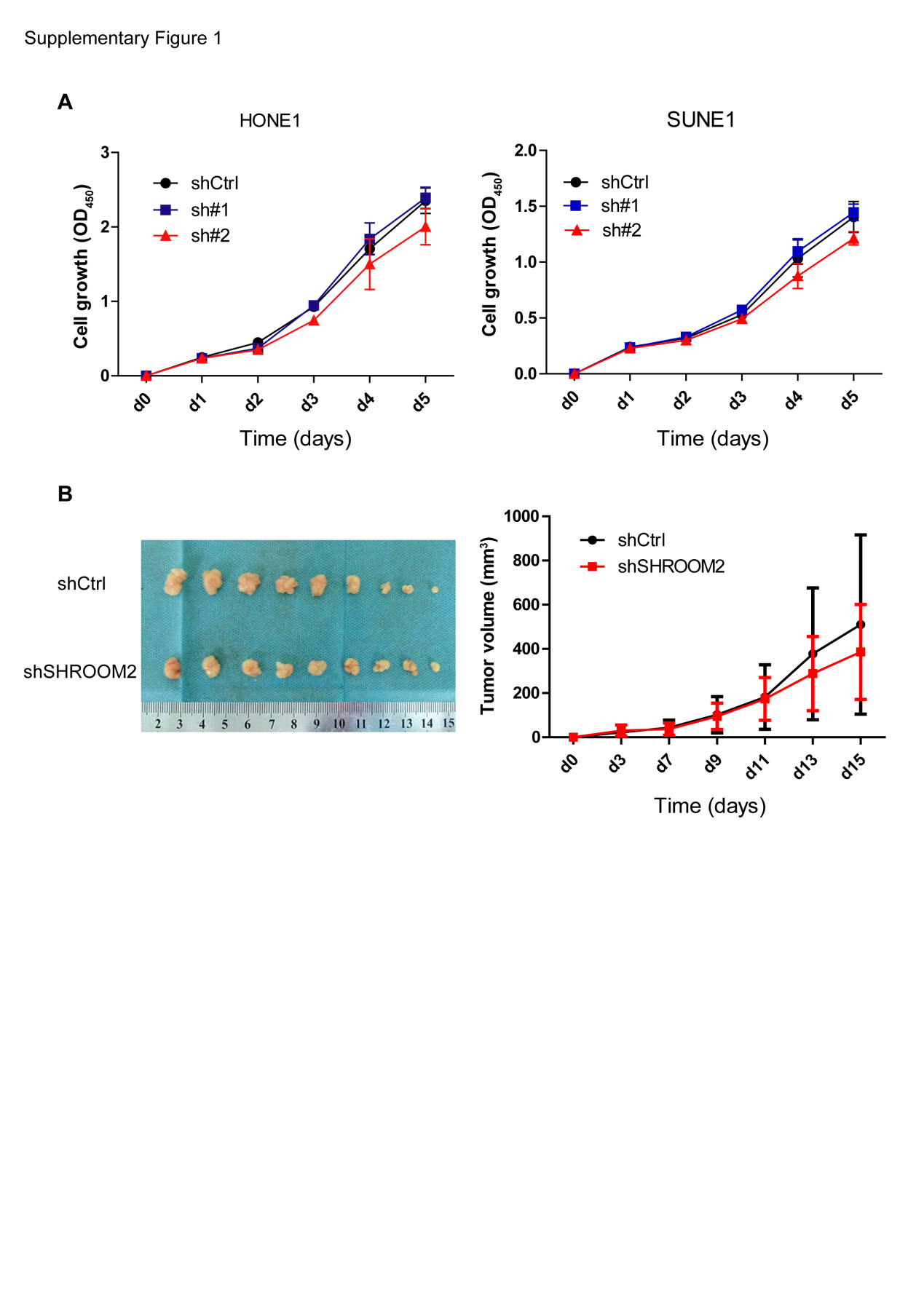

Supplement: Supplementary file 1 — Supplementary Figure [file 41419_2019_1325_MOESM1_ESM.docx]
